# Development and validation of a nomogram based on preoperative variables for predicting recurrence‐free survival in stage IA lung adenocarcinoma

**DOI:** 10.1111/1759-7714.15099

**Published:** 2023-10-04

**Authors:** Jiaxi Xu, Hui Zeng, Guochao Zhang, Renda Li, Zhenlong Yuan, Jingyu Ren, Yufei Huang, Fangzhou Ren, Hao Zhang, Kailun Fei, Feiyue Feng, Fengwei Tan

**Affiliations:** ^1^ Department of Thoracic Surgery, National Cancer Center–National Clinical Research Center for Cancer–Cancer Hospital Chinese Academy of Medical Sciences and Peking Union Medical College Beijing China; ^2^ Department of Immunology and National Key Laboratory of Medical Molecular Biology, Institute of Basic Medical Sciences Chinese Academy of Medical Sciences (CAMS) and Peking Union Medical College Beijing China; ^3^ Department of Medical Oncology, National Cancer Center–National Clinical Research Center for Cancer–Cancer Hospital Chinese Academy of Medical Sciences and Peking Union Medical College Beijing China

**Keywords:** lung adenocarcinoma, non‐small cell lung cancer, preoperative blood parameters, sublobar resection

## Abstract

**Background:**

This study aimed to establish a nomogram for predicting risk of recurrence and provide a model for decision‐making between lobectomy and sublobar resection in patients with stage IA lung adenocarcinoma.

**Methods:**

Patients diagnosed with stage IA lung adenocarcinoma (LUAD) between December 2010 and October 2018 from Cancer Hospital Chinese Academy of Medical Sciences were included. Patients were randomly assigned to training and validation cohorts, accounting for 70% and 30% of the total cases, respectively. We collected laboratory variables before surgery. Univariate and multivariate analyses were performed in the training cohort to identify variables significantly associated with recurrence‐free survival (RFS) which were subsequently used to construct a nomogram. Validation was conducted in both cohorts. A receiver operating characteristic curve was used to determine the optional cutoff values of the scores calculated from the nomogram. Patients were then divided into low‐ and high‐risk groups. Survival was performed to determine if the nomogram could guide the operation method.

**Results:**

A total of 543 patients were included in this study. Gender, albumin level, carcinoembryonic antigen level and cytokeratin‐19‐fragment level were included in the nomogram. In both cohorts, the nomogram stratified the patients into high‐ and low‐risk groups in terms of RFS. In particular, there was a significant difference in RFS between lobectomy and sublobar resection in the high‐risk group.

**Conclusions:**

Gender, albumin level, carcinoembryonic antigen level and cytokeratin‐19‐fragment level are valuable markers in predicting recurrence and can guide surgical practice in patients with stage IA LUAD.

## Graphical abstract

The process of constructing and validating of nomogram. Patients were stratified into high and low risk groups. After that the subgroup analysis was conducted.

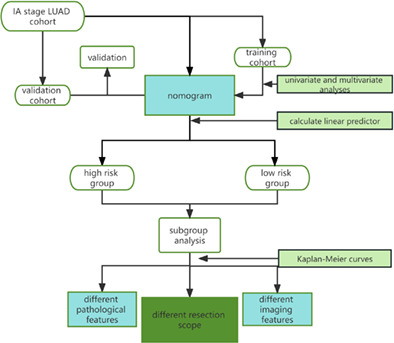

## INTRODUCTION

Lung cancer is one of the most frequently diagnosed cancers and the leading cause of cancer‐related deaths worldwide, with an estimated 2 million new cases and 1.76 million deaths per year.^1^ There are two main types of primary lung cancer: non‐small cell lung cancer (NSCLC) and small cell lung cancer (SCLC). NSCLC accounts for approximately 85% of all new lung cancer cases.^1^ Lung adenocarcinoma (LUAD) is the most common lung cancer subtype,^2^ accounting for 50% of all lung cancer cases.^2^, ^3^ Early diagnosis and surgical intervention of stage IA NSCLC has significantly improved the long‐term survival of patients with lung cancer.^4^ However, 10% of patients with stage IA LUAD suffer disease recurrence in 5 years, composing a unique subpopulation with a high recurrence risk.^5^

It has been reported that segmentectomy is connected with a higher risk of local recurrence.^6^ Given the higher risk of local recurrence in patients who receive segmentectomy, identifying the subpopulation with a high recurrence risk using a prediction model before surgery and performing lobectomy for them is promising.^7^

In recent years, some serum blood cell parameters, such as the neutrophil‐lymphocyte ratio (NLR) and platelet‐lymphocyte ratio (PLR), have been reported to be associated with the prognosis of many kinds of malignant tumors.^8^, ^9^ Levels of preoperative platelets and albumin level are also related to the prognosis of cancer.^10^, ^11^, ^12^ Serum tumor biomarkers, such as carcinoembryonic antigen (CEA), carbohydrate antigen125 (CA125), cytokeratin‐19‐fragment (Cyfra21‐1), squamous cell carcinoma (SCC), and neuron‐specific enolase (NSE), are also widely used in the diagnosis and prognostic monitoring of lung cancer.^13^, ^14^, ^15^ Thus, serum parameters are valuable prognostic models in lung cancer diagnosis, recurrence, metastasis, and various treatment outcomes. However, the performance of preoperative blood parameters to predict recurrence in patients with stage IA LUAD have not been extensively examined. A prediction model composed of preoperative parameters is urgently needed to identify the high of recurrence subpopulation in patients with stage IA LUAD.

Here, we conducted a study to construct a nomogram using preoperative parameters to identify high risk of recurrence subpopulation in patients with stage IA LUAD. We evaluated the performance of this model in a real‐world patient cohort from a high‐volume cancer center. We also discovered that this model may be valuable to select a better surgery method for the high risk of recurrence subpopulation.

## METHODS

### Patients, clinical characteristics, and variables

This study was approved by the Ethics Committee of the National Cancer Center/Cancer Hospital, Chinese Academy of Medical Sciences. Patients who received radical surgery for lung cancer pathologically and were diagnosed with stage IA LUAD according to the eighth edition of AJCC TNM classification for lung cancer from December 2010 to October 2018 were screened for eligibility. All patients underwent surgery with negative surgical margins and lymph node dissection. Patients who received adjuvant or neoadjuvant therapies were excluded. Laboratory variables were collected 1 week before the surgery. After surgery, follow‐up of the patients was performed every 3 months for the first and second year, every 6 months for the third to fifth years, and then annually thereafter until recurrence or the last follow‐up. The endpoint was recurrence. Recurrence‐free survival (RFS) was defined as the time between the date of surgery and the date of the first recurrence and/or distant metastasis or the last follow‐up. The follow‐up ended in December 2021. Cases were divided into training and validation cohorts. The training cohort was used to build a prediction model, and the validation cohort was used to validate the model. Information of the clinical characteristics and laboratory data, including age, gender, smoking history and white blood cell (WBC), red blood cell (RBC), neutrophil count (Neut), lymphocyte count (Lymph), monocyte count (Mono), platelet count (PLT), hemoglobin (Hb), lactate dehydrogenase (LDH), albumin (ALB), NSE, CEA, CA125, Cyfra21‐1, and SCC levels, were extracted from the patients' electronic medical records. We also calculated the systemic immune‐inflammation index (SII), PLR, and NLR as following: SII = PLT × Neut /Lymph, NLR = Neut/Lymph, PLR = PLT/ Lymph.^7^, ^8^, ^9^ Images of CT were estimated by at least two radiologists independently for radiological features. Lung nodules only consisting of ground‐glass opacity (GGO) were categorized as pure nodules, while nodules only consisting of solid part were categorized as pure solid nodules. The rest of the nodules comprising GGO and solid parts were simultaneously considered as partial‐solid pulmonary nodules. Pathological subtypes were classified according to the lung adenocarcinoma classification of WHO. Lung adenocarcinoma was categorized into lepidic LUAD, acinar LUAD, papillary LUAD, micropapillary LUAD and solid LUAD.

### Construction of prediction model

We constructed receiver operating characteristic (ROC) curves to determine the optional cutoff values of all variables based on samples in the training cohort. The values of maximum joint sensitivity and specificity on the ROC plot were defined as the recommended cutoff value. Continuous variables were converted into dichotomous variables by their optimal cutoff. Both univariate and multivariate analyses were used to analyze variables affecting RFS, and variables significantly associated with RFS were included in the multivariate analysis. A nomogram was created using variables significantly associated with RFS in the multivariate analysis.

### Evaluation of performance of the prediction model

The performance of the nomogram was evaluated using discrimination and calibration. The model's calibration was determined by combining two validation methods. One validation (bootstrap method with 1000 resamples) to obtain Harrell's concordance index (C‐index), and the validation cohort was used to further validate the prediction model. Calibration curves were plotted to calibrate the model. We also used decision curve analysis (DCA curves) to compare our model and single clinical variables. The scores of each patient were calculated by nomogram. After that, a receiver operating characteristic curve was constructed to determine the optional cutoff value of the scores. Patients were divided into low‐ and high‐risk groups using the cutoff value of score. The log‐rank test was used to compare RFS between the high‐ and low‐risk groups. After that, we compared recurrence difference in different subgroups (different surgery scope, different clinicopathological characteristics and different tumor stages) All statistical test levels were considered statistically significant at *p* ≤ 0.05.

## RESULTS

### Baseline of training and validation cohort

After screening and quality control, a total of 543 patients were included in this study, with 380 patients assigned to the training cohort and 163 to the validation cohort. The baseline characteristics of the whole, training and validation cohorts are summarized in Table 1. The median and mean follow‐up times of the training cohort were 1818 and 1678 days, and the validation cohort were 1854 and 1735 days. By the end of this study, 42 (11.0%) patients in the training cohort and 17 (10.4%) patients in the validation cohort experienced recurrence.

**TABLE 1:** Clinicopathological characteristics and blood variables of the cohort.

			Status		
		Total *n* = 543	No recurrence *n* = 484 (89.1)	Recurrence *n* = 59 (10.9)	*p*‐value
WBC (%)	Low	370	328 (67.8)	42 (71.2)	0.659
	High	173	156 (32.2)	17 (28.8)	
Neut (%)	Low	200	176 (36.4)	24 (40.7)	0.568
	High	343	308 (63.6)	35 (59.3)	
Lymph (%)	Low	332	293 (60.5)	39 (66.1)	0.480
	High	211	191 (39.5)	20 (33.9)	
Mono (%)	Low	101	86 (17.8)	15 (25.4)	0.158
	High	438	398 (82.2)	44 (74.6)	
SII (%)	Low	60	51 (10.5)	9 (15.3)	0.273
	High	483	433 (89.5)	50 (84.7)	
PLR (%)	Low	227	203 (41.9)	24 (40.7)	0.890
	High	316	281 (58.1)	35 (59.3)	
NLR (%)	Low	139	118 (24.4)	21 (35.6)	0.081
	High	404	366 (75.6)	38 (64.4)	
Hb (%)	Low	250	224 (46.3)	26 (44.1)	0.783
	High	293	260 (53.7)	33 (55.9)	
RBC (%)	Low	374	336 (69.4)	38 (64.4)	0.458
	High	169	148 (30.6)	21 (35.6)	
PLT (%)	Low	77	66 (13.6)	11 (18.6)	0.322
	High	466	418 (86.4)	48 (81.4)	
LDH (%)	Low	134	113 (23.3)	21 (35.6)	0.054
	High	409	371 (76.7)	38 (64.4)	
ALB (%)	Low	393	342 (70.7)	51 (86.4)	0.009*
	High	150	142 (29.3)	8 (13.6)	
NSE (%)	Low	314	290 (60.5)	24 (40.7)	0.005*
	High	224	189 (39.5)	35 (59.3)	
CEA (%)	Low	156	148 (30.6)	8 (13.6)	0.006*
	High	386	335 (69.4)	51 (86.4)	
CA125 (%)	Low	369	334 (69.9)	35 (59.3)	0.104
	High	168	144 (30.1)	24 (40.7)	
SCC (%)	Low	146	132 (27.6)	14 (23.7)	0.642
	High	392	347 (72.4)	45 (76.3)	
Cyfra211 (%)	Low	409	372 (78.0)	37 (62.7)	0.014*
	High	127	105 (22.0)	22 (37.3)	
T classification	T1a	129	127 (27.4)	2 (3.6)	<0.001*
	T1b	256	230 (49.6)	26 (47.3)	
	T1c	134	107 (23.1)	27 (49.1)	
Gender (%)	Female	297	274 (56.6)	23 (39.0)	0.012*
	Male	246	210 (43.4)	36 (61.0)	
Smoking history (%)	Low	327	294 (67.9)	33 (55.9)	0.078
	High	165	139 (32.1)	26 (44.1)	
Age (%)	≤65	400	359 (74.2)	41 (69.5)	0.437
	>65	143	125 (25.8)	18 (30.5)	
Family (%)	No	366	323 (66.7)	43 (72.9)	0.380
	Yes	177	161 (33.3)	16 (27.1)	
Resection scope (%)	Lob	425	374 (77.3)	51 (86.4)	0.132
	Sublobar	118	110 (22.7)	8 (13.6)	
Imaging features (%)	Pure solid	139	103 (21.5)	36 (64.3)	<0.001*
	Partial solid	236	216 (45.1)	20 (35.7)	
	Pure ground‐ glass	160	160 (33.4)	0 (0.0)	
Pathological features (%)	Lepidic	88	86 (19.9)	2 (3.9)	<0.001*
	Acinar	293	270 (62.4)	23 (45.1)	
	Papillary	74	59 (13.6)	15 (29.4)	
	Solid	23	15 (3.5)	8 (15.7)	
	Micropapillary	3	3 (0.7)	3 (5.9)	

Abbreviations: ALB, albumin; CEA, carcinoembryonic antigen; Hb, hemoglobin; LDH, lactate dehydrogenase; Lymph, lymphocytes; Mono, monocytes; Neut, neutrophils; NLR, neutrophil‐lymphocyte ratio; NSE, neuron‐specific enlase; PLR, platelet‐lymphocyte ratio; PLT, platelets; RBC, red blood cells; SCC, squamous cell carcinoma; SII, systemic immune‐inflammation index; WBC, white blood cells.

### Assignment of continuous variables to dichotomous variables

The optimal cutoff values for each laboratory data were as follows: WBC = 6.74 × 10^9^/L, Neut = 3.085 × 10^9^/L, Lymph = 2.015 × 10^9^/L, Mono = 2.085 × 10^9^/L, SII = 242.06, NLR = 1.43, PLR = 116.62, Hb = 140.5 g/L, RBC = 4.915 × 10^12^/L, PLT = 178.5 × 10^9^/L, LDH = 153.5 U/L, ALB = 47.35 g/L, NSE = 13.345 ng/mL, CEA = 1.465 ng/mL, CA125 = 11.48 U/mL, SCC = 0.55 ng/mL, and Cyfra21‐1 = 2.795 ng/mL. All the continuous variables in both the training and validation cohorts were then converted into dichotomous variables denoted as “high” and “low” based on comparison with the cutoff value. The comparison of the two baselines of different cohorts is shown in Table 2.

**TABLE 2:** The comparison of the two baselines of different cohorts.

Characteristics		Train cohort	Validation cohort	*p*‐value
Status	Recurrence	42	17	0.830
	Nonrecurrence	338	146	
WBC	High	128	45	0.160
	Low	252	118	
Neut	High	243	100	0.570
	Low	147	63	
SII	High	342	141	0.029
	Low	38	22	
NLR	High	284	120	0.790
	Low	96	43	
PLR	High	226	90	0.320
	Low	154	73	
Lymph	High	145	66	0.610
	Low	235	97	
Mono	High	318	124	0.037
	Low	62	39	
Hb	High	208	85	0.580
	Low	172	78	
RBC	High	254	120	0.120
	Low	126	43	
PLT	High	324	142	0.570
	Low	56	21	
LDH	High	287	122	0.870
	Low	93	41	
ALB	High	97	53	0.100
	Low	283	110	
NSE	High	155	69	0.650
	Low	223	91	
CEA	High	271	115	0.820
	Low	108	48	
CA125	High	125	43	0.070
	Low	252	117	
SCC	High	272	120	0.570
	Low	105	41	
Cyfra21‐1	High	85	42	0.370
	Low	292	117	
Age	>65	102	41	0.680
	≤65	278	122	
Smoking	Yes	121	44	0.330
	No	226	101	
Gender	Male	179	67	0.200
	Female	201	96	
Family history	Yes	126	51	0.670
	No	254	112	

Abbreviations: ALB, albumin; CEA, carcinoembryonic antigen; Hb, hemoglobin; LDH, lactate dehydrogenase; Lymph, lymphocytes; Mono, monocytes; Neut, neutrophils; NLR, neutrophil‐lymphocyte ratio; NSE, neuron‐specific enlase; PLR, platelet‐lymphocyte ratio; PLT, platelets; RBC, red blood cells; SCC, squamous cell carcinoma; SII, systemic immune‐inflammation index; WBC, white blood cells.

### Construction and validation of the nomogram

Due to our study only focusing on preoperative blood parameters, all variables in Table 1, except T stage and extent of resection, were calculated using univariate and multivariate Cox proportional hazard regression models. Gender, CEA, CA125, and ALB were discovered to be significantly associated with RFS by univariate analysis and were input into the multivariate analysis. Finally, all four factors above were also discovered to be associated with RFS by multivariate analysis and were included in the construction of the nomogram (Table 3).

**TABLE 3:** Selected factors in the training cohort for building the nomogram via univariate and multivariate analyses.

Variables	Univariate		Multivariate	
	HR (95% CI)	*p*	HR (95% CI)	*p*‐value
WBC	0.8268 (0.4335, 1.584)	0.569		
Neut	0.928 (0.5055, 1.704)	0.81		
Lymph	0.7159 (0.3795, 1.351)	0.302		
Mono	0.5944 (0.2932, 1.205)	0.149		
SII	0.9055 (0.3561, 2.302)	0.835		
PLR	1.28 (0.6923, 2.367)	0.431		
NLR	0.7409 (0.3928, 1.398)	0.354		
RBC	1.337 (0.728, 2.456)	0.349		
Hb	1.409 (0.767, 2.588)	0.269		
PLT	0.8922 (0.3977, 2.001)	0.782		
LDH	0.7246 (0.3836, 1.369)	0.321		
ALB	0.3908 (0.1539, 0.9923)	0.0481*	0.3669 (0.144, 0.9347)	0.035*
NSE	1.765 (0.9745, 3.197)	0.06		
CEA	3.345 (1.316, 8.5)	0.011*	2.674 (1.03, 6.9451)	0.0434*
CA125	1.456 (0.798, 2.655)	0.221		
SCC	0.9686 (0.5062, 1.854)	0.923		
Cyfra21‐1	2.901 (1.597, 5.271)	<0.001*	2.6916 (1.478, 4.9013)	0.0012*
Age (>65)	1.154 (0.6038, 2.207)	0.664		
Gender (male)	2.2216 (1.197, 4.104)	0.011*	2.0303 (1.087, 3.7937)	0.0264*
Smoking	1.596 (0.8767, 2.907)	0.126		

Abbreviations: ALB, albumin; CEA, carcinoembryonic antigen; Hb, hemoglobin; LDH, lactate dehydrogenase; Lymph, lymphocytes; Mono, monocytes; Neut, neutrophils; NLR, neutrophil‐lymphocyte ratio; NSE, neuron‐specific enlase; PLR, platelet‐lymphocyte ratio; PLT, platelets; RBC, red blood cells; SCC, squamous cell carcinoma; SII, systemic immune‐inflammation index; WBC, white blood cells.

* indicates thevariables with significant differences were marked.

**FIGURE 1:**
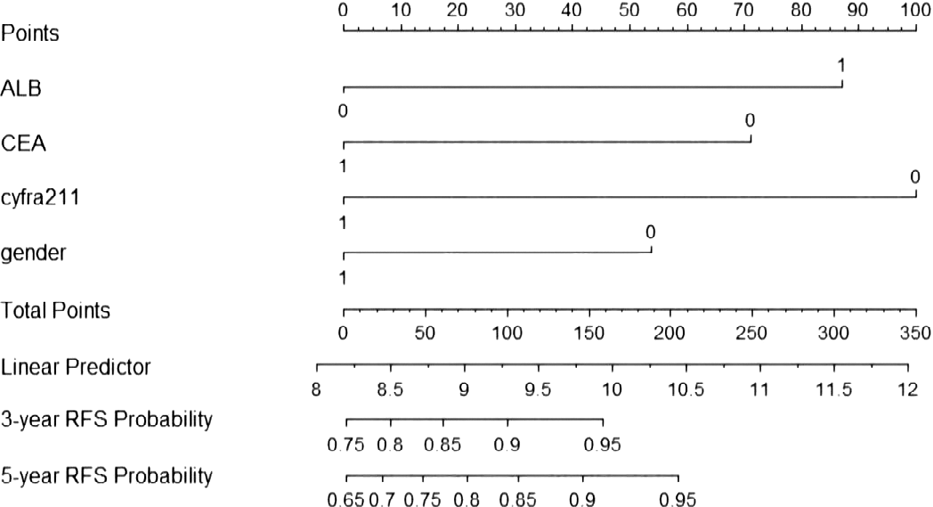
Nomogram based on univariate and multivariate analyses.

**FIGURE 2:**
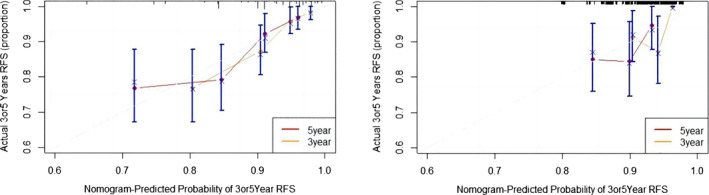
Calibration curve of the training (left) and validation cohorts(right).

**FIGURE 3:**
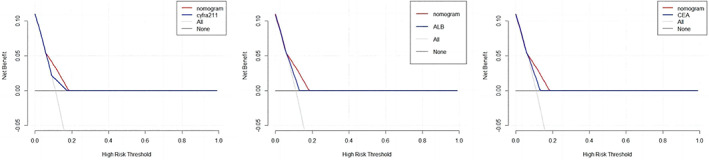
Decision curve analysis indicates better efficiency of our model than the other single indicators at thresholds between 0.1 and 0.2 (cyfra21‐1 left, ALB middle, CEA right).

The nomogram is presented in Figure 1. In the training cohort, the C‐index was 0.74 after 1000 times of internal self‐sampling. The C‐index of 0.619 in the validation cohort showed that the nomogram had good accuracy and application. The slope of the calibration curve was close to the reference line, indicating that the model could better predict the RFS of patients. The calibration curve was constructed. The slope of the calibration curves was close to the reference line, indicating that the model could predict the RFS rate of samples (Figure 2). Finally, the DCA curves showed that our model had better efficiency than the other single indicators in the nomogram at thresholds between 0.1 and 0.2 (Figure 3).

### Prediction value of the nomogram

**FIGURE 4:**
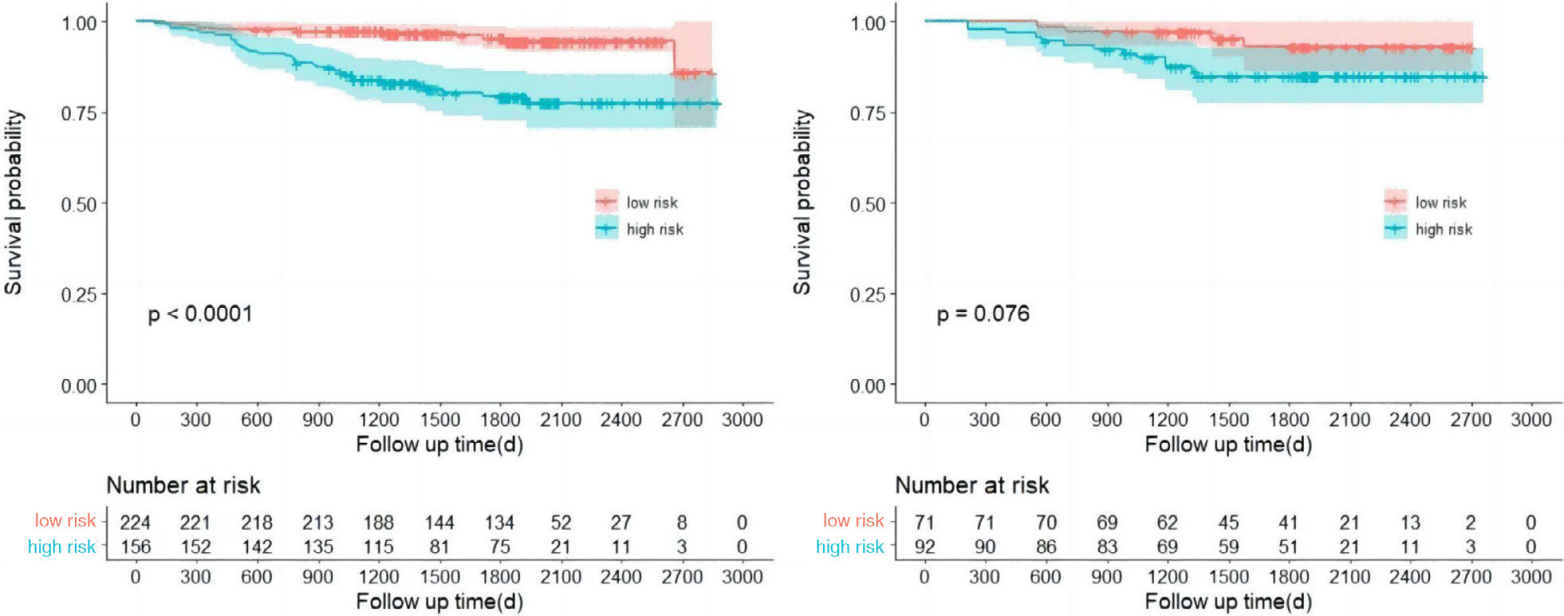
Kaplan–Meier curves of recurrence‐free survival (RFS). Stratified by the nomogram in the training (left) and validation (right) cohorts.

The linear predictor of each patient (the scores of each patient) was calculated from the nomogram and the cutoff value was determined as 10 using the ROC method. A statistically significant difference in RFS between the high‐risk group (score < 10) and low‐risk population (score ≥ 10) was observed in the training cohorts (*p* < 0.001, respectively), as shown in Figure 4. The survival curves of the high‐ and low‐risk group in the validation cohort were well separated (Figure 4, right panel), suggesting a good prediction value in the validation cohort. However, the *p*‐value was slightly higher than the significant level (*p* = 0.076) which was probably due to the limited size of the validation cohort.

### Value of selection method of surgery of the nomogram

**FIGURE 5:**
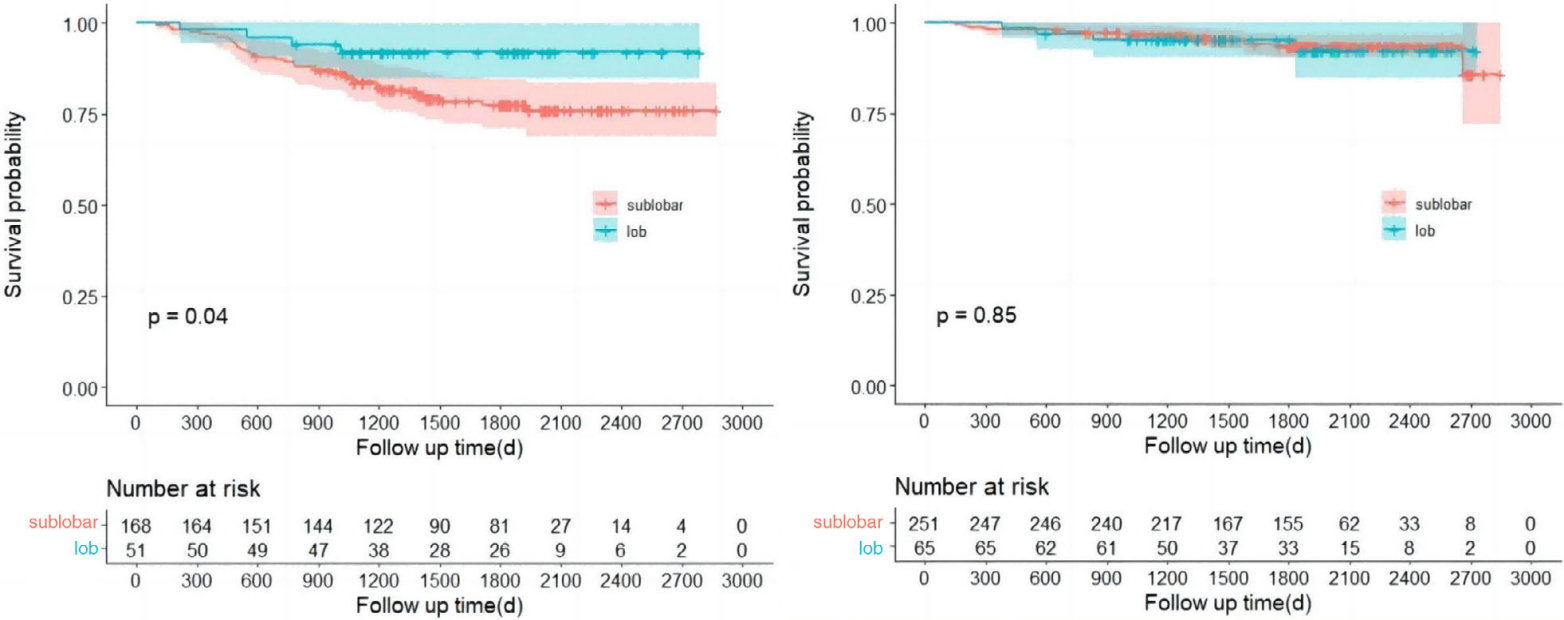
Kaplan–Meier curves of recurrence‐free survival (RFS) for lobar or sublobar resection in the high‐risk (left) or low‐risk (right) group.

We also observed statistically significant differences of RFS between patients who received lobectomy and sublobar resection in the high‐risk subgroups (*p* = 0.04, Figure 5, left panel). In patients with high risk of recurrence, the 5‐year RFS was 7.5% for patients who received lobectomy, and 21.4% for those who received sublobar resection. In the low‐risk group, lobectomy and sublobar resection yielded comparable RFS (*p* = 0.85, 5‐year RFS 5.9% for lobectomy vs. 6.1% for sublobar resection). These results indicate that lobectomy may be a better choice in terms of reducing recurrence for patients identified as high risk by the nomogram. By evaluation using preoperative parameters, this nomogram may help to achieve improvement in surgical decision‐making.

### Association of pathological and imaging features with risks predicted by the nomogram

**FIGURE 6:**
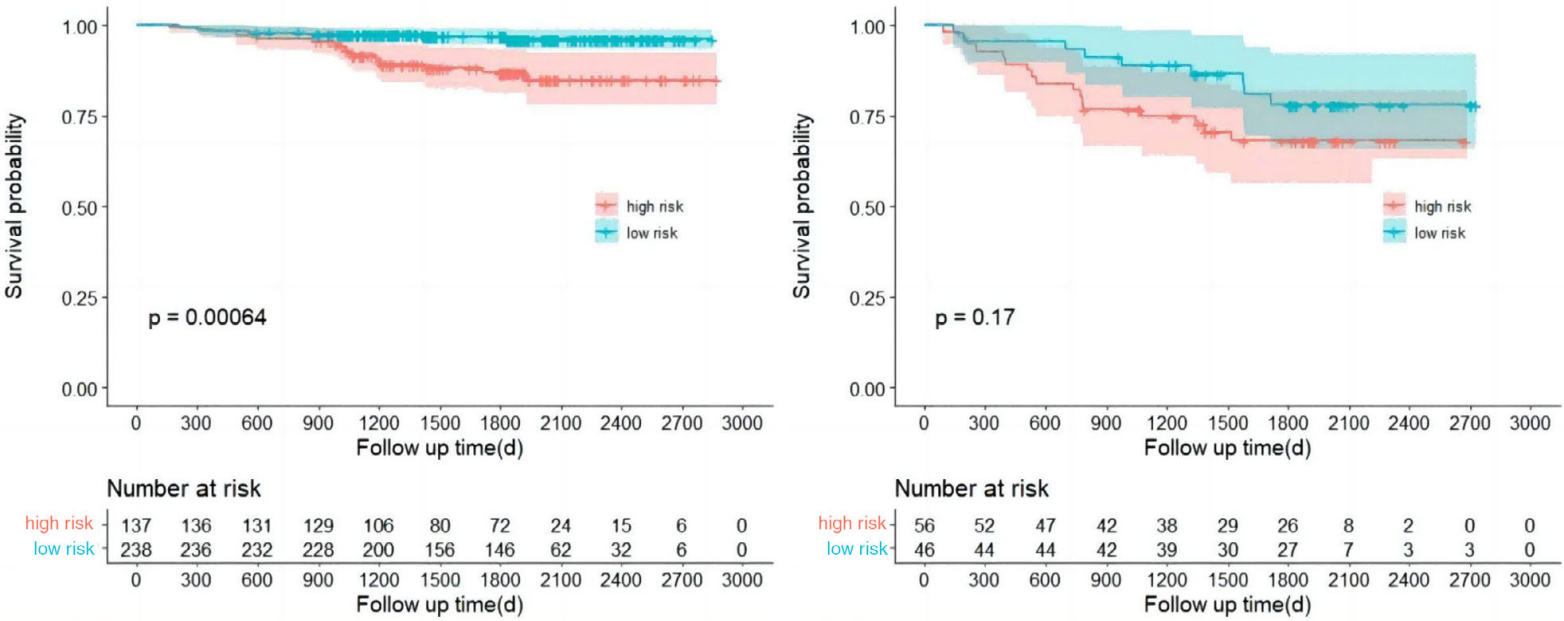
Kaplan–Meier curves of recurrence‐free survival (RFS) for risk stratification, lepidic lung adenocarcinoma (LUAD), acinar LUAD and papillary LUAD (left), and micropapillary LUAD and solid LUAD (right).

**FIGURE 7:**
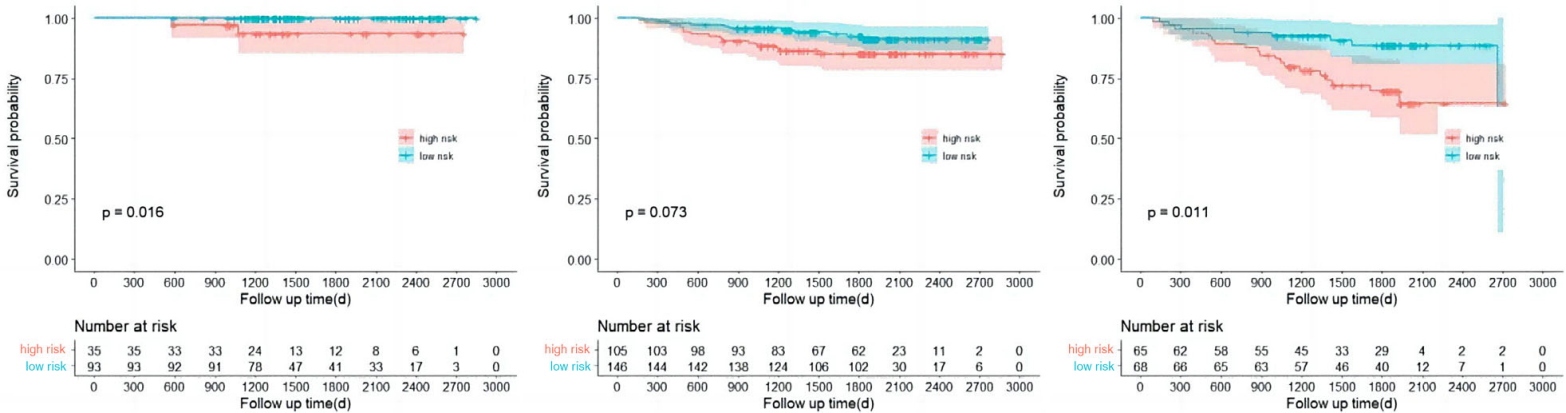
Kaplan–Meier curves of recurrence‐free survival (RFS) for risk stratification and the groups of the different tumor stages. T1a (left), T1b (middle), and T1c (right).

**FIGURE 8:**
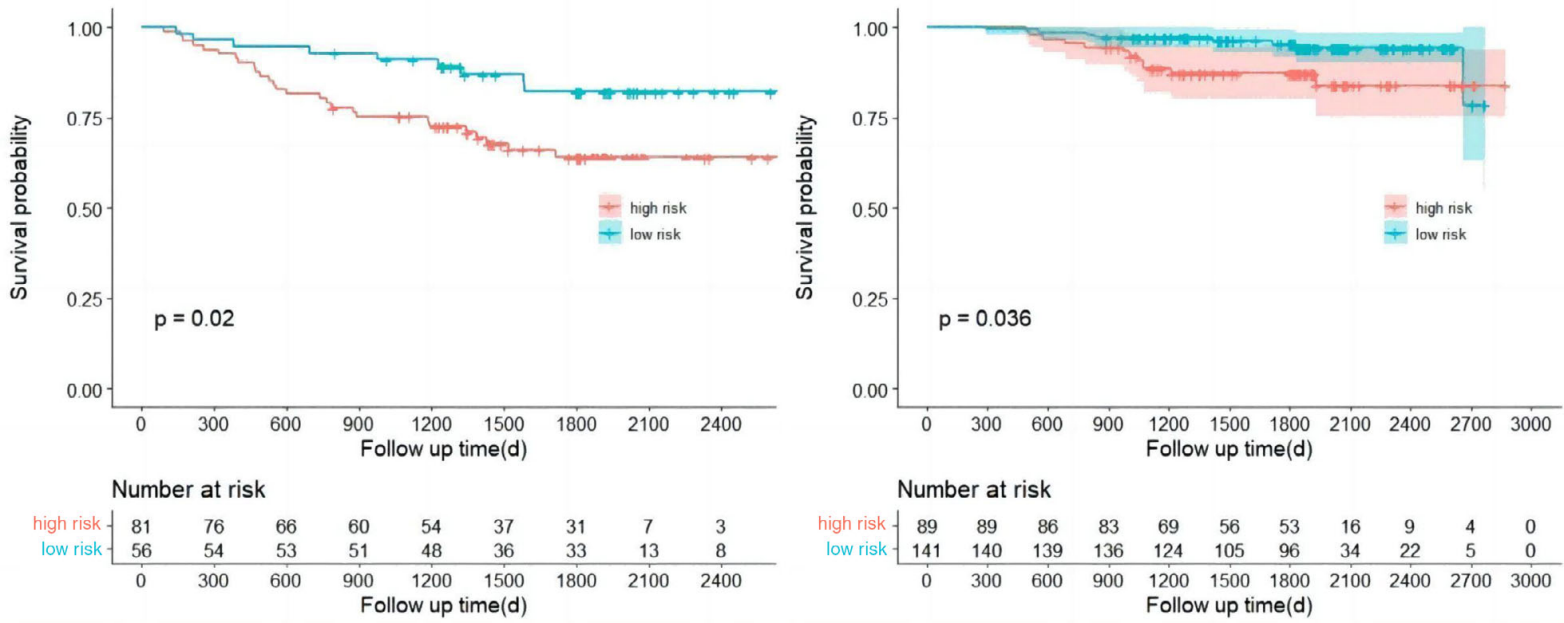
Kaplan–Meier curves of recurrence‐free survival (RFS) for risk stratification, group of pure solid lung nodules (left), partial‐solid pulmonary nodules (right).

We further examined the prediction value of the nomogram in subgroups of stage IA patients with different pathological and imaging features. We found the RFS of high‐risk patients was significantly worse than that of the low‐risk patients in subgroup analysis of different pathological features (*p*‐value), tumor size (*p*‐value), and imaging features (*p*‐value), as shown in Figures 6, 7, and 8, respectively. These findings suggest the prognostic value of our model may be independent of the tumor size, imaging and pathological features.

## DISCUSSION

In this study, we mainly used preoperative blood parameters to build a model which not only had prognostic value but could also provide a reference for thoracic surgeons when choosing resection before surgery. Patients at higher risk of recurrence may benefit from lobectomy resection, while the same effect could be achieved in sublobar resection patients at lower risk as surgeons can save more lung tissue. This finding makes great sense in patients of greater age or those with pulmonary dysfunction.

Our cohort had some superiorities. First, it focused only on stage IA LUAD patients with a huge sample size, while previous studies concentrated on locally advanced disease or metastasis. Moreover, this model is the first prediction model to predict recurrence in stage IA LUAD patients only structured by preoperative blood parameters. Patients with early‐stage LUAD experience local recurrence and distant metastasis due to micrometastasis that cannot be detected by current technology.^16^, ^17^ Moreover, previous studies have focused more on postoperative blood parameters, which have led to a delay in the discovery of recurrence. Furthermore, preoperative blood parameters are generally acquired before surgery, which makes the use of the model universal. Therefore, we explored the preoperative hematological variables and established an effective model to predict the recurrence of stage IA LUAD, which fills the research gap.

Nowadays, the indication for sublobar resection is still controversial. In terms of T1aN0 disease, it is suggested that the rate of local recurrence patients with sublobar resection is higher than lobectomy.^18^ On the other hand, there have also been studies which have suggested that segmentectomy is noninferior and superior to lobectomy with regard to overall survival.^7^ Thus, a prediction model is urgently needed to discern the stage IA LUAD patients who may benefit from sublobar resection. In this study, we analyzed the influence of different surgical scopes in different groups. We revealed that patients at higher risk could benefit more from lobectomy than sublobar resection. Interestingly, there was no significant difference in the recurrence risk between lobectomy and sublobar resection in the low‐risk‐patient group. This phenomenon suggests that preoperative blood parameters may be valuable prognostic factors when choosing different resection scopes. However, our study only focused on stage IA adenocarcinoma, and it is difficult for a surgeon to verify the stage and pathological information of the tumor before surgery. Therefore, we suggest that the surgical scheme should be determined after obtaining the result of an intraoperative frozen biopsy.

In this study, we constructed a nomogram to distinguish stage IA patients who had a high risk of recurrence. We believe that serum markers play an important role in lung cancer treatment, but these markers are underestimated in the preoperative prediction of stage IA lung adenocarcinoma. As a tumor marker with a prognostic role in lung adenocarcinoma, CEA is conveniently detected during the postoperative period and has been widely used in clinical practice. However, in a previous study, the recommended pretreatment evaluation indicator of the NCCN guidelines for the management of NSCLC issued did not contain CEA.^19^ On the other hand, Cyfra21‐1 is widespread in epithelial cells, and the malignant transformation of epithelial cells activates protease, which accelerates the degradation of keratin, thereby resulting in the release of Cyfra21‐1 into the blood. It has been reported that Cyfra21‐1 expression is higher in patients with squamous cell carcinoma than in patients with glandular cancer and SCLC.^20^, ^21^ However, in our study, Cyfra21‐1 also performed well in the prognosis of stage IA lung adenocarcinoma. This phenomenon was also observed in another lung adenocarcinoma study.^22^ The value of Cyfra21‐1 in the prognosis of stage IA lung adenocarcinoma still requires further molecular analysis.

Serum albumin has also been shown to have prognostic value. Some studies have reported that albumin is associated with the prognosis of early‐stage lung cancer.^12^, ^23^ However, previous studies have focused mainly on patients with advanced lung cancer.^24^ Due to the close connection between albumin and nutrition, the level of albumin has been commonly measured in the treatment of advanced cancer to estimate patient prognosis.^25^, ^26^ Obviously, patients with stage IA lung adenocarcinoma seldom suffer from malnutrition. In this study, we discovered that albumin level is an important preoperative parameter for forecasting recurrence, but the potential mechanism by which the serum albumin level influences recurrence has not been adequately elucidated. There is also no evidence that improving the serum albumin level will reduce the probability of recurrence of stage IA lung adenocarcinoma.

In this study, we combined three blood parameters and confirmed that the prognostic value of the combination of these parameters was higher than each one alone which illustrates the necessity of the establishment of our nomogram. Due to the absence of specific markers, the prognosis of LUAD is always difficult. However, our nomogram accomplished this task, thus meeting the demand from clinical practice.

Furthermore, we analyzed the difference of RFS in two risk groups with stratification by clinicopathological characteristics. The results showed that in the analysis of three subgroups, the trend of the probability of recurrence between the high‐ and the low‐risk groups could be distinguished according to Kaplan–Meier curves. Although the *p*‐value was sometimes not statistically significant, we considered this condition to be a result of the absence of samples. In conclusion, we consider that the prognostic value of preoperative blood parameters in stage IA lung adenocarcinoma may be independent of the tumor size, imaging features and pathological features.

There are several issues to be considered with the findings of our study. First, we only focused on stage IA lung adenocarcinoma, while other studies mostly focused on NSCLC at all stages. Moreover, we only used the basic blood parameters that are essential for diagnosis and surgery. This indicates that our nomogram will be cost‐efficient and does not require the collection of other parameters. However, there are also other limitations. First, due to the retrospective characteristics of the study, the existence of bias is inevitable. Second, the parameters included in the nomogram may be affected by many factors. Third, our study was a single institution study, and multicenter studies are needed in the future.

In conclusion, in this study, we discovered that gender, ALB, CEA, and Cyfra21‐1 are valuable prognostic variables in stage IA lung adenocarcinoma, which not only can help predict recurrence, but can also provide guidance for surgical practice. It should be emphasized that CEA, Cyfra21‐1, ALB monitoring during the preoperative period will provide valuable prognostic information for patients with IA stage lung adenocarcinoma. Higher levels of CEA and Cyfra21‐1 are connected with recurrence. On the contrary, higher levels of ALB and being female are protective factors. We suggest that patients with high levels of preoperative level of CEA and Cyfra21‐1 or low ALB level will benefit from lobectomy.

## AUTHOR CONTRIBUTIONS

Jiaxi Xu, Zeng hui, Guochao Zhang responsible for data analysis and composing the article, Renda Li Zhenlong Yuan, Jingyu Ren, Yufei Huang, Fangzhou Ren, Hao Zhang, Kailun Fei responsible for collecting data. Fengwei Tan and Feiyue Feng responsible for review, editing and proofreading.

## CONFLICT OF INTEREST STATEMENT

The authors declare no conflict of interest.
